# Using Artificial Intelligence for Assistance Systems to Bring Motor Learning Principles into Real World Motor Tasks

**DOI:** 10.3390/s22072481

**Published:** 2022-03-23

**Authors:** Koenraad Vandevoorde, Lukas Vollenkemper, Constanze Schwan, Martin Kohlhase, Wolfram Schenck

**Affiliations:** Center for Applied Data Science (CfADS), Faculty of Engineering and Mathematics, Bielefeld University of Applied Sciences, 33619 Bielefeld, Germany; lukas.vollenkemper@fh-bielefeld.de (L.V.); constanze.schwan@fh-bielefeld.de (C.S.); martin.kohlhase@fh-bielefeld.de (M.K.)

**Keywords:** motor learning, motor skill learning, assistance system, artificial intelligence, machine learning, pose estimation, action recognition, human motion analysis

## Abstract

Humans learn movements naturally, but it takes a lot of time and training to achieve expert performance in motor skills. In this review, we show how modern technologies can support people in learning new motor skills. First, we introduce important concepts in motor control, motor learning and motor skill learning. We also give an overview about the rapid expansion of machine learning algorithms and sensor technologies for human motion analysis. The integration between motor learning principles, machine learning algorithms and recent sensor technologies has the potential to develop AI-guided assistance systems for motor skill training. We give our perspective on this integration of different fields to transition from motor learning research in laboratory settings to real world environments and real world motor tasks and propose a stepwise approach to facilitate this transition.

## 1. Introduction

Motor learning is a broad concept that can be defined as any experience-dependent improvement in motor performance [1]. The first well-investigated principle of motor learning is called motor sequence learning and investigates in detail how we perform several motor actions after one another with the aim of improving the speed and accuracy of a sequence of actions [2]. The second well-known principle is called motor adaptation. This principle allows us to adjust our movements and make them robust to external perturbations [3]. For instance, when walking on different surfaces or terrain, we automatically adjust our walking pattern according to the properties of the surface. Motor skill learning is an extension of motor learning, as it allows us to perform a motor task of interest better, faster, or more accurate than before [4] and requires extended practice over hours, weeks, or months [5]. Behavioral experiments have provided great insights into motor learning at the behavioral and neural levels [1,6]. However, surprisingly few applications exist so far that target the two well-studied principles of motor learning to improve motor skill learning. A possible reason for this is the large gap that remains between what we know from conventional laboratory experiments about motor learning principles and motor learning in dynamic natural environments [7].

Concurrently, human motion analysis techniques have improved drastically over the last decades. Especially in the field of artificial intelligence, huge progress has been achieved. For instance, it has become possible to accurately track human motion in dynamic natural surroundings; to estimate human body and hand poses in RGB images, depth images and RGB-depth images [8,9,10,11,12]; to detect the objects and tools that are used or are visible in the surroundings [13]; to estimate object poses [8,14] and to recognize human actions [15,16,17]. All these developments have reached high accuracy with the progress made in machine and, especially, deep learning [18,19]. In this paper, we will focus on techniques from the machine learning family, of which deep learning is a subpart. These techniques have the potential to bridge the existing gap between the insights from laboratory experiments and the natural environment in which motor learning normally takes place [7]. Since every technique has its own limitations and advantages, the complementary use of different analysis techniques is often recommended. This is also the case for different sensors that can be used to characterize motion or provide feedback. A trend exists to assist human motion with sensors and algorithms for different motor skills, ranging from sport applications [20] to music education [21] to surgery [22] to industry tasks [23,24,25,26]. However, so far, these applications rarely implement insights from motor learning to optimize the learning process.

One important step is to scale up the motor learning principles from laboratory experiments to 3D real world problems. Currently, complex motor behavior is largely left unexplored, since most studies are performed in well-controlled lab environments [27]. Gradually increasing the complexity of the studied motor behavior becomes possible with improved observation and analysis techniques. We call this the bottom-up approach, since this approach starts from fundamental motor learning principles and gradually increases the complexity of the experimental motor tasks to approach closer to everyday motor skills. In contrast, one can start from a motor skill of interest and gradually divide it into individual components of motor learning, here called the top-down approach. Assistance systems to train a specific motor skill already exist, but optimizing the learning process with knowledge from motor learning is a new approach. We think that both approaches are necessary to close the gap that exists between the laboratory knowledge about motor learning and applications to efficiently train motor skills.

**Figure 1:**
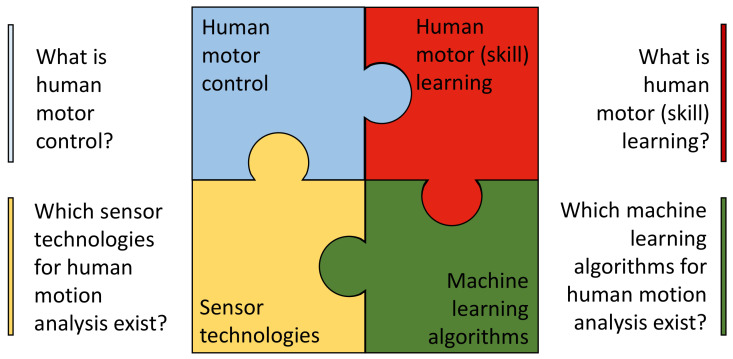
Transferring human motor learning principles to real world applications requires the integration of several research domains.

The scope of this review is to discuss how classical motor learning and motor control research can transition from the laboratory to a real world environment to enhance motor skill learning of real world motor tasks. The review starts with an introduction to motor control in Section 2 (Figure 1, left top) and an overview of two well-known motor learning principles and their relation to motor skill learning in Section 3 (Figure 1, right top). Subsequently, Section 4 discusses a selection of human motion analysis algorithms (Figure 1, right bottom), and Section 5 focuses on sensor technologies (Figure 1, left bottom). Finally, Section 6 integrates motor learning research with human motion analysis algorithms and sensor technology. In this section, a two-fold approach is proposed to bridge the gap that exists between motor learning principles studied in laboratory environments and real world complex motor skills. The bottom-up approach shows how, starting from well-known motor learning principles, the complexity can be increased to investigate motor skill learning, while the top-down approach starts from a motor skill of interest and quantifies its performance by assessing the relevant motor learning parameters. In the long term, these approaches can help to improve motor skill training by human assistance systems.

## 2. Human Motor Control

**Figure 2:**
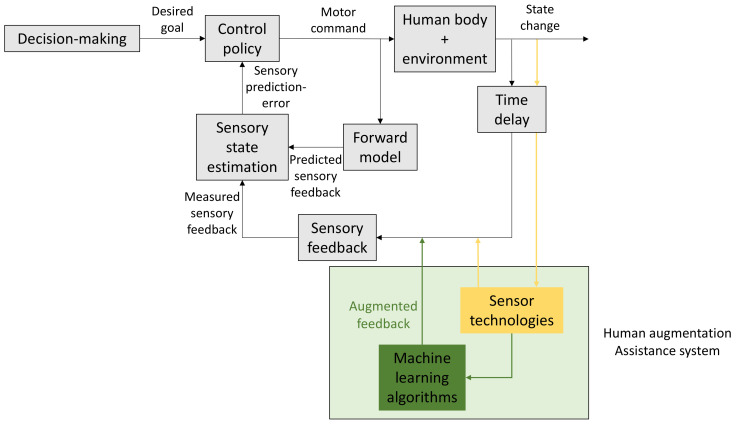
Human motor control scheme (adapted from Shadmehr et al. [6]), extended with sensor technologies and augmented feedback to design an AI-guided assistance system for motor skill training.

In the discipline of motor control, we study how organisms make accurate goal-directed movements [28]. A motor command is sent to the muscles of our body (Figure 2). This results in a specific movement trajectory and an end position of the activated body parts (state change), which can be observed by the sensory system with a short delay. In parallel, an efference copy of the motor command is used by the forward model to predict the movement trajectory and the end position before it occurs. The difference between the predicted state and the observed state is the sensory prediction error. This error is used by the control policy to generate the next motor command. Together, the feedback and feedforward loop allow efficient and accurate control of the muscles.

Motor control can, to a large extent, occur without much cognitive effort; imagine, for example, the daily actions that we undertake, like drinking, eating, standing and walking. All these actions can be done without requiring our full cognitive capacity. However, also, cognitive decision-making can influence human motor control by selecting the desired movement goal (Figure 2) [29]. At some points, motor control requires additional cognitive effort; imagine walking across a road with heavy traffic. Briefly, you need to time very well when you start crossing the road and increase the vigor of your walking compared to how fast you usually do. Additional cognitive effort is also necessary when we learn to make new movement patterns, when we learn to improve our movement accuracy or when we learn to improve our movement timing. If we want to improve our movement timing, for example, temporarily, we can decide to increase the cost for incorrect timing. Later, we can decide to practice more on movement accuracy, and a reweighting occurs with an increased cost for an inaccurate movement or more weight on reaching the desired visuospatial goal.

In addition, in this review, we expand the motor control scheme (colored elements in Figure 2) to visualize how human assistance systems can improve human motor learning. Besides the human sensory system, sensor technology can play a similar role to register and quantify observable state changes of the human body. Different machine learning algorithms can be used to analyze the obtained sensor data. These analyzed sensor data can be fed back to the user as augmented feedback, which complements its own body sensory feedback. Sensor data can also be provided directly to the user without extensive processing. The additional information can have an impact on the decision-making process. The augmented feedback can result in an altered weighting of each decision criteria and, hence, result in a different movement goal.

## 3. Human Motor Learning

The motor control scheme (Figure 2) showed how organisms make accurate goal-directed movements. In motor learning, we study how organisms, with practice, can improve the motor performance of these goal-directed movements. In this section, we give a brief overview of a selection of two principles of motor learning. For comprehensive reviews discussing motor learning principles, we refer to the following excellent reviews in motor learning: H. E. Kim et al., 2021; Krakauer et al., 2019; Shadmehr et al., 2010; Wolpert et al., 2011 [1,7,28,30].

### 3.1. Motor Sequence Learning

Motor sequence learning occurs when separate movements are integrated into a unified and coordinated sequence of actions through practice [31]. This sequence of actions can either be several discrete actions or several continuous and (partially) overlapping actions. For example, preparing a cup of tea are several discrete steps, while a smash in badminton are several continuous actions of the whole body [1]. Performance improvement in motor sequence learning occurs, often in the time–domain of the movement, as an improved reaction time or a faster movement time for a sequence of actions. In the laboratory, sequence learning is often studied using a finger tapping task, during which buttons on a keyboard should be pressed in a specific order [32]. The simplest finger tapping task requires the repeated execution of a short sequence of 4–6 elements [1]. Each finger is represented by a digit, such that each digit indicates which finger should press the underlying button (e.g., index: 1, middle: 2, ring: 3 and little: 4). The sequence is provided to the participants at the start of the task, and the goal of the task is to execute the sequence as accurate and as fast as possible. With practice, sequential action execution becomes faster, more accurate and largely automatic [1,32].

**Figure 3:**
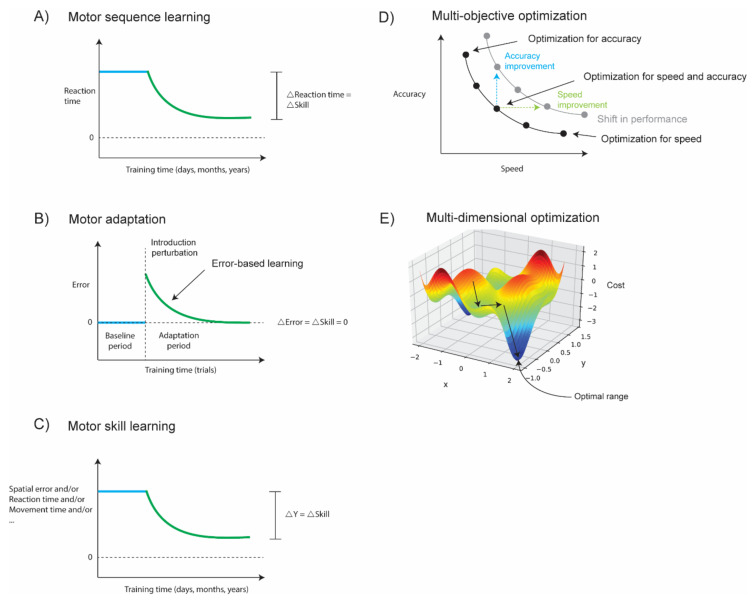
Motor sequence learning and motor adaptation are two well-known motor learning principles, which are also active during motor skill learning. (**A**) Motor sequence learning. Only a single parameter is optimized, usually the reaction time or response time. Optimization lasts after training. (**B**) Motor adaptation. No skill learning, only recalibration of the current motor performance to a new changed parameter (e.g., string tension) that induced a sudden error. Only a single parameter is optimized, usually angular error or spatial error. Error-based learning (green curve) is the process of reducing errors. (**C**) Motor skill learning. Optimization that lasts after training. The parameter(s) to optimize can be spatial error, reaction time, movement time or many others. (**D**) Multiple-objective optimization deals with the optimization of more than 1 objective simultaneously; often, these objectives are conflicting. In motor skill learning, conflicting objectives could be optimized for speed and for accuracy. (**E**) Multidimensional optimization is optimization that deals with many dimensions. In motor skill learning, optimization can depend on variables like the reaction time, movement speed, accuracy, body posture, limb coordination, predicted movement and many more. Expert demonstration of the motor skill provides a reference for the desired range of the different variables. This can help to find the right direction for the optimization problem faster or to take bigger steps during the optimization (learning curve schemas in (**A**–**C**) adapted from Sternad (2018) [74]).

The most-used paradigm to study sequence learning is the Serial Reaction Time Task. During this task, participants have to respond to a visual cue as fast as possible by pressing the corresponding button with their finger. Alternative options are arm reaching to buttons or foot presses. The response should be made only after the visual cue appears. The sequence (S) of target appearance has a fixed order, which is learned through practice. The fixed sequential order of targets is often alternated with a random (R) order of targets to correct for changing reaction times to random targets (e.g., by changing attention). Sequence-specific learning is calculated as the S–R difference of the reaction times [1]. Performance improvements of the reaction time occur with practice in an exponentially decreasing way (Figure 3A), but improvements also occur in between practice sessions [33,34].

An important explanation for sequence learning is the grouping of individual elements into chunks. As learning progresses, the chunks become larger and eventually result in an entire sequence. The length of the chunks and their structure may depend on the working memory capacity [35]. Chunking might also explain why the generalization of sequence learning occurs. Generalization is the transfer of sequence learning to untrained but similar sequences or to a different effector (e.g., the other hand). Chunking is the grouping of elements or the representation of order rather than the motor action itself [36]. This order representation might thus help to speed up the learning process of a similar order with the same hand or to speed up the execution of the same order with the other hand.

Different aspects of sequence learning are learned explicitly or implicitly. For example, Wong and colleagues showed that both a random sequence and a fully explicit sequence were executed faster with extended practice. The gradual performance improvement did not differ for both sequences, suggesting that this gradual improvement was sequence-independent and that no sequence-specific implicit learning occurred. However, an immediate improvement in response time existed for the fully explicit sequence, reflecting explicit sequence knowledge [37].

### 3.2. Motor Adaptation

Motor adaptation occurs when movements are adjusted to perturbations or changes in the environment [3]. For instance, when humans walk on different surfaces or terrains, or with rested or tired muscles, they automatically adjust their walking pattern to the specific conditions. A recent study with quadruped robots showed that robots also require a real-time motor adaptation module to successfully walk on various terrains [38]. Another example is, when lifting different objects, humans automatically adjust their grip according to the weight of the object [39,40,41]. In motor adaption, learning is triggered by errors, often in the spatial domain of the movement, for instance, as the compensation for a walking error, a lifting error or a reaching error. In lab experiments, human motor adaptation is most typically studied for upper limb movements using a tablet computer or a robot. Participants are instructed to perform sequential arm reaching movements on the tablet with the arm made invisible. The task is to move a cursor on the monitor from a start position towards a target. At a specific point in time, a perturbation is introduced as a rotation of the cursor with respect to the hand motion (Figure 3B). Participants should adapt to this perturbation by moving their hand in the opposite direction of the rotation (reduce error in Figure 3B). This experimental paradigm to study motor adaptation is called visuomotor rotation. Motor adaptation can also be studied with a forcefield paradigm on a robot. Again, participants are instructed to make arm reaching movements to reach targets on a monitor. Instead of a rotational perturbation, a force perturbation is executed by the robot on the participant’s hand. The participant can adapt to the perturbation by opposing the perturbing force, which is called forcefield adaptation. Besides these often-used paradigms to study the motor adaptation of upper limb movement, alternative paradigms exist to study the motor adaptation of gait [42,43,44], speech [45,46,47] and eye movement [48,49,50,51,52,53].

Motor adaptation can be dissociated in underlying components, which can be observed with adjusted versions of the typical motor adaptation experiments. A first way to dissociate motor adaptation is by the dependence on cognitive processes. Motor adaptation can occur either unconsciously (i.e., implicit) or with cognitive control (i.e., explicit) [54]. The explicit process is the easiest to understand. For example, in a visuomotor rotation experiment with a 30-degree counterclockwise rotation, a participant may decide to aim somewhat to the right of the target (e.g., 20 degrees). In that case, we say that the participant’s explicit strategy equals 20 degrees. The most optimal explicit strategy to counter a 30-degree perturbation would be to aim 30 degrees clockwise with respect to the target. However, most participants do not use aiming angles, which exactly match the perturbation size, since they are not informed about all details of the perturbation. Several experimental paradigms exist to assess explicit adaptation during motor adaptation experiments [54,55,56].

A participant adapts unconsciously or implicitly as well. As a result, the actual reaching angle is bigger than the aiming angle. The difference between the actual reaching direction and the explicit aiming direction equals the amount of implicit adaptation. The driving factor for implicit adaptation is the mismatch between the predicted sensory feedback and the observed (and perturbed) sensory feedback, called the sensory prediction error. The sensory prediction error is used in the motor control scheme, but the exact link between implicit adaptation and motor control is not clear. It appears to be involved in updating both the control policy and the forward model (Figure 2) [29]. A participant automatically minimizes this sensory prediction error by moving the arm in the opposite direction of the perturbation. Implicit adaptation is a slow process, while explicit adaptation is faster [54]. Depending on the task parameters (perturbation size, targets, reaction time, etc.), the relative contribution of implicit versus explicit adaptation is different [57,58,59,60].

Additionally, motor adaptation can be assessed depending on the presence (or absence) of reward or punishment. Learning from reward is also called reinforcement learning. Several adaptation paradigms have been designed to assess the effect of reward on motor adaptation [61,62,63,64,65]. Reward has a positive effect on the retention of motor adaptation, while punishment enhances the learning rate [62].

### 3.3. Motor Skill Learning

Motor skill learning allows to perform a motor task better, faster or more accurately than before [4] and requires extended practice over hours, weeks or months [5]. For instance, learning to play badminton (or tennis) well requires several years of training and can be considered as an example of motor skill learning.

Remember the process of motor sequence learning, where a specific sequence of actions is learned to be executed faster, more fluently or more accurately with practice. One example for badminton could be the training of a specific stroke such as smashing. During training, a novice player learns to smash by combining different subelements or postures. If a badminton player smashes during a game, these subelements cannot be recognized anymore, since no clear boundaries exist between the individual subelements. This example shows that sequence learning is one way to contribute to the complex process of motor skill learning.

This contrasts with motor adaptation, which only investigates the recalibration of the existing task performance to a changed condition [3] and is often possible within a few practice trials [58,66] or even within a single trial [67]. For instance, learning to play badminton (or tennis) well requires several years of training (i.e., motor skill), but getting used to playing badminton with a new racket sometimes only requires a few training days or, with new strings on the existing racket, only a couple of strokes (i.e., motor adaptation). In addition, since the task performance does not improve compared to the baseline performance (Figure 3B, ΔError = ΔSkill = 0), motor adaptation could, according to its definition, not be considered as a process contributing to motor skill learning [4,68,69]. Nevertheless, motor adaptation enables that the forward and inverse model (or control policy) (Figure 2) remain calibrated for various external changes, which ensures the robustness of the movement. Without motor adaptation, a new motor performance level can be reached, but a small change in any relevant parameter could result in complete motor skill failure. In addition, the process of error reduction or cost optimization are inherent to both motor adaptation (Figure 3B, green curve) and motor skill learning (Figure 3C left top, green curve). In motor adaptation, the process of error-based learning (Figure 3B, green curve) is essential to recalibrate the performance back to the baseline level after a perturbation, while, in motor skill learning, it is an important process to reach a new performance level. Therefore, we argue here that the study of motor adaptation does contribute to the understanding of motor skill learning through the process of error-based learning.

However, the optimization problem is more complex in motor skill learning (Figure 3C) than in motor adaptation paradigms, which we know from laboratory experiments (e.g., visuomotor rotation and forcefield adaptation). Therefore, in motor skill learning, much more practice is required to reach an optimal solution range [70,71]. For instance, if we use the badminton example again, the optimization occurs in a high number of dimensions. One typically first learns the basic strokes of the game: the serve, dropshot, smash, clear and lob. For each of these strokes, the player must control their posture at different steps in the stroke, control the timing of the motion, control eye–hand–body coordination and control the racket orientation. During the game itself, the player should control their footwork and monitor and predict the state of the shuttle and the opponent, while, at the same time, making tactical choices between different strokes and directions to play the shuttle. Each of the steps described above requires optimization from years of training to reach an expert performance level of the motor skill. Instead, when only replacing the strings of the badminton racket, the only optimization necessary is the error reduction to control for the change in shuttle velocity induced by the increased string tension [72].

Morehead and Orban de Xivry [29] recently proposed how the weight of each component of the loss function could be determined by cognitive decision-making in visuomotor adaptation. In other words, cognitive decision-making defines the weights for the different objectives in multi-objective optimization. Multi-objective optimization typically deals with the optimization of multiple conflicting objectives [73]. In motor learning, two clearly conflicting objectives are speed and accuracy of the movement (Figure 3D). This typically results in a trade-off between the different objectives, and the speed–accuracy trade-off is a well-known one. We argue here that the same applies to motor skill learning, with the number of objectives (Figure 3D) and the number of dimensions (Figure 3E) of the optimization problem higher than for visuomotor rotation experiments, one reason for this being the control of the entire body in a three-dimensional space compared to upper limb motor control in only two dimensions. It is plausible that increasing the number of objectives and dimensions increases the amount of practice required for successful optimization.

Training a motor skill requires many training sessions with different exercises, these exercises are useful to allow a person to focus only on a few of their errors and to optimize the performances for these errors (i.e., reducing the dimensions of the optimization problem). For instance, one could focus only on the posture errors during the serve in racket sports (i.e., accuracy improvement, blue arrow in Figure 3D); alternatively, one could focus on improving the speed of the movement on the court (i.e., speed improvement, green arrow in Figure 3D). A shift in the speed–accuracy trade-off can be defined as an increased motor skill performance [75]. Another element of training is the demonstration of the desired motion by an experienced person before the exercise is executed by an unexperienced person. This will allow the unexperienced person to become better aware of the error they are making and correct for it. This visual demonstration can serve as a reference for the desired action that can be imitated, and this way, the optimization in the enormous multidimensional space (Figure 3E) can proceed faster [76]. Sometimes, it is not only a visual demonstration that can function as a reference; for instance, when learning to play a new piano piece, the teacher will play the song before the pupil starts playing. Here, the memory of the desired sound can serve as an alternative reference for the desired finger movements. By dividing a motor skill into subskills and by demonstrating the desired behavior, training sessions can be designed to reduce the overall error complexity (or dimensionality) of the motor skill to be trained and bring specific errors into focus.

Most motor learning and motor skill learning research is conducted in laboratory settings with strictly controlled movement parameters, whereas real world motor learning is typically very variable, as optimization occurs in many dimensions, given that the human body has many degrees of freedom and given that many solutions exist to solve the same motor task. This contradiction shows that it is necessary to create new experimental paradigms that closer match the real behavior and environment with its intrinsically high variability. This is where we can benefit from the recent developments in machine learning algorithms and sensor technologies for human motion analysis.

## 4. Machine Learning Algorithms for Human Motion Analysis

In this section, we describe a selection of machine learning algorithms useful for analyzing human motion. First, we discuss dimensionality reduction techniques to transform motion data to a low-dimensional space that captures the dimensions with the highest variability in the data. Then, the algorithms are divided into four different categories according to purpose: pose estimation, action classification, motion prediction and motion comparison. The representation of the motion is given by skeleton data (relative joint positions and angles) captured with inertial measurement units or image sequences recorded from RGB, depth or RGB–depth camera(s). For teaching a motor skill, a teaching system needs to be able to detect the actual pose, determine the according motion (motion classification) and determine the difference to the desired motion (motion comparison) to instruct the novice how to correct their motion. The prediction of motion could help to predict a mismatch with a desired motion at an early stage. Finally, we discuss how developments in robot motor learning can be useful to test new hypotheses about human motor (skill) learning.

**Figure 4:**
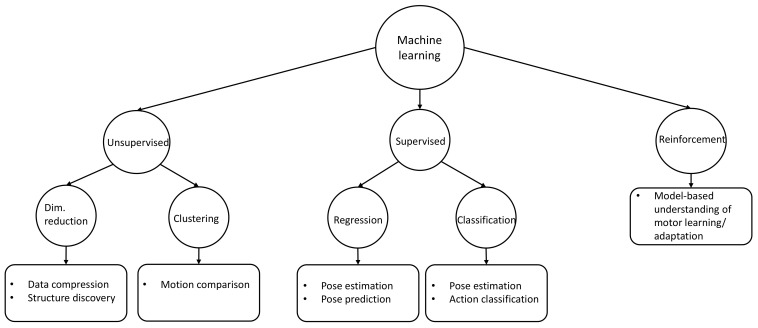
Machine learning categorization and some example attributions of human motion analysis problems. This schema should not be considered as a strict separation or as the only possible existing combinations, but rather, it shows the most frequently occurring categories.

Machine learning algorithms can be divided into different categories: unsupervised, supervised and reinforcement learning (Figure 4). In unsupervised learning, the machine learning algorithm is used to find structures inside the data without prior knowledge. Two subclasses of unsupervised learning are clustering and dimensionality reduction. Clustering algorithms try to discover clusters in data based on a distance measure. For motion comparison, for example, two motions are considered to be the same when the distance of the joint positions is smaller than a given threshold. In supervised learning, the training data consists of an input and a desired output. The task of the machine learning algorithm is to learn the relevant features from the training data while generalizing for unknown data. Supervised learning can be differentiated into regression (continuous output) and classification (discrete output). Pose estimation, for example, can be treated as a regression or a classification problem. Finally, machine learning algorithms can make use of artificial neural networks to achieve higher accuracies if combined with powerful computing. The choice of the machine learning algorithm for a human motion analysis problem often depends on the data used for training. In reinforcement learning, the decision of the machine learning algorithm is evaluated after each prediction step. The system gets penalized for bad predictions and rewarded for good predictions. The overall task of the algorithm is to maximize the reward function.

Databases exist for different types of motions (e.g., drinking, eating, walking or even taking a selfie [77]); different body parts (e.g., hand [14,78] or body [79]); different sensor types (e.g., Vicon system [80], inertial measurement units [81], RGB video [82] or RGB–depth [83]) or for human–object interactions [84].

### 4.1. Structure Discovery and Data Compression by Using Dimensionality Reduction Techniques

Dimensionality reduction techniques can transform high-dimensional data to a low-dimensional space. Their use is beneficial for human motion datasets, which contain many measurement trials, measurement variables or combine multiple measurement techniques. Dimensionality reduction can help to discover structure in the data [85,86,87], to compress the data [88] or to enable easier visualization [89]. It can be applied directly or after pose estimation but can also be used as an action recognition method by itself [90]. Nguyen and Holmes [91] present ten practical tips for the effective application of dimensionality reduction techniques. At first, it can seem intimidating to select the correct dimensionality reduction technique among the many techniques that exist (for a comparative overview of the techniques, see Van Der Maaten et al. [92]). Therefore, the first of the ten tips is the choice of the dimensionality reduction technique based on the input data. For instance, nonlinear dimensionality reduction techniques can better deal with complex nonlinear data, which could be favorable in real world data that presents itself as nonlinear manifolds [92]. They do preserve local interactions well, but for preserving the global data structure, linear techniques are the better choice [91]. The core idea of dimensionality reduction techniques is to find the intrinsic dimensionality of the data, which is the minimum number of parameters required to account for the properties of the data [93]. The most well-known unsupervised linear reduction technique is principal component analysis (PCA). PCA constructs a low-dimensional representation of the data by searching for the linear basis of reduced dimensionality with maximal variance in the data [92]. PCA has been applied for technique analyses in sports (e.g., skiing: Federolf et al. [94] and Gløersen et al. [95]), for data compression of natural motion (e.g., hand motion: Lin et al. [96]), for the comparison of motions among different experience levels (e.g., race walking: Donà et al. [97]) or conditions (e.g., ergonomic assessment during a lifting task: Sadler et al. [98]). This shows some of the many possibilities that dimensionality reduction offers for a human motion analysis. Besides linear reduction techniques, nonlinear techniques also offer great potential in human motion analysis. For example, Uniform Manifold Approximation and Projection (UMAP) has been used to analyze soccer players’ skills [99], and deep autoencoders have been used to find a representation of movement in a latent feature space [100]. The movement that the different neurons in this latent space represent can be visualized by using dynamic movement primitives [101] as an additional hidden layer.

### 4.2. Motion Comparison with Clustering

After dimensionality reduction, a useful next step is clustering the data. Clustering algorithms divide the data into a number of clusters (groups, subsets and categories) [102]. A formal definition for a cluster does not exist, but it could be described as a set of entities that are alike, and entities from different clusters are not alike. Data from the same cluster are similar to each other, while data from different clusters are dissimilar from one another [102]. For a motion analysis, one could group the data according to experience level (novice vs. intermediate vs. expert); according to applied techniques or strategies or according to movement patterns. For instance, Marques et al. [87] used a two-stage unsupervised clustering approach to identify 13 different swimming patterns in zebrafish larval movements. They used a custom-developed density-based clustering method. In sports, clustering can be used to compare players based on a set of attributes. An example is the work by Lopes and Tenreiro Machado [99] where this approach was used for assessing different soccer player styles. Another example from sports shows how clustering can be used to extract temporal behavior of a specific movement. Ghasemzadeh and Jafari [103] used k-means clustering on kinematic data from the hip, shoulder and wrist to divide a baseball swing motion into specific groups of frames that were similar. From these groups, they analyzed the coordination of the movement and determined if a sequence of actions from the hip, arm and shoulder was performed with good or bad timing of the key events. In surgery, unsupervised temporal clustering was applied to chunk a surgical procedure into clinically relevant tasks [104]. After comparing four different temporal clustering algorithms, they concluded that the hierarchical aligned cluster analysis method outperformed the others, with an average segmentation accuracy of 88.0%. These examples show that, together with dimensionality reduction techniques, clustering methods can bring structure into a complex dataset without the need for a labor-intensive-labeled dataset.

All clustering algorithms need a notion of similarity to find groups. We show recent articles presenting different similarity measures that are or could be used for cluster analysis (Table 1). We provide the input data type and the task solved. As we can see, most similarity measures need 3D joint positions as inputs. The exceptions are 3D curves, quaternions and RGB videos. Three-dimensional curves are used to represent a line in space as a sequence of direction changes [105]. Quaternions are used to represent rotations in three-dimensional space. A motion sequence is translated into a set of rotations for each limb and timeframe [106]. RGB video was used by Park et al. [107] but could be grouped with 3D joint positions, as one of the first steps in their approach was to pose a 3D estimation onto the video data.

Similarity measures between different motions are important to human motor learning experiments, because they can be used as a measure of conformance of a novice action with an expert execution. Alternatively, for one subject, we could compare the beginning of a movement sequence to the end of the movement sequence in order to assess whether learning occurred for this subject. We showed that significant progress has been made for similarity measures during the last couple of years (Table 1) and argue that this should be used by motor control researchers when explicit measures of movement quality are not available.

### 4.3. Pose Estimation

Moeslund et al. [118] defined pose estimation as the process of estimating the configuration of the underlying kinematic or skeletal articulation structure of a person. This usually means estimating 2D/3D coordinates for a set of joints in some simplified human skeleton. Since the successful use of convolutional neural networks (CNNs) for tasks like object recognition (AlexNet) [19], the use of CNNs dominates the state-of-the-art methods in nearly any image-based task [119]. Especially in pose estimation from images or videos, the use of convolutional neural networks was beneficial. For in-depth overviews on pose estimation with neural networks, we refer to References [120,121,122] or the most recent from Zheng et al. [123]. In this study, we focus on the most prominent techniques. Human pose estimations can be separated using 2D and 3D methods. A brief overview of these two approaches is given in the following two subsections.

#### 4.3.1. Pose Estimation in Two Dimensions

**Figure 5:**
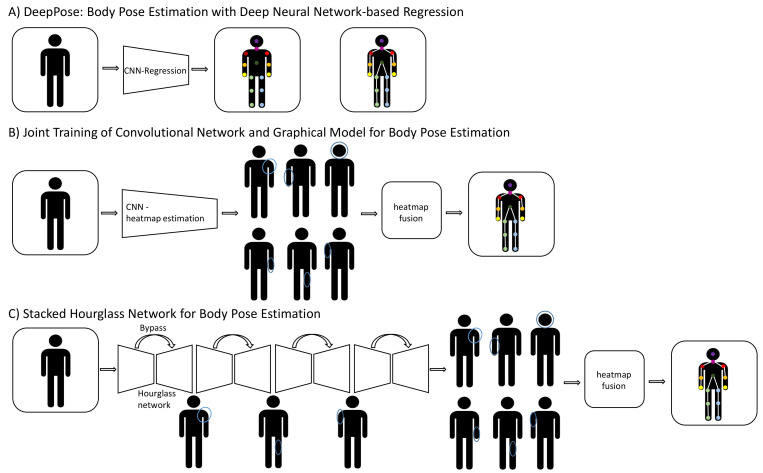
Overview of 2D human pose estimation approaches with deep learning. (**A**) Deep learning-based regression predicts the key points as 2D coordinates directly on the image. The 2D pose is the connected graph of the predicted key points. (**B**) Deep learning-based pose detection generates a set of body part heatmaps that are fused to generate the 2D pose estimation. (**C**) Architecture of the stacked hourglass network introduced by Newell et al. [125]. Each individual hourglass module consists of convolutional layers (encoding), followed by deconvolutional layers (decoding). The maximum activations across each predicted heatmap are the final estimated pose.

For 2D pose estimations with neural networks, two approaches can be distinguished: pose regression and pose detection (Figure 5). In the regression-based approach, a CNN predicts onto the input image the 2D coordinates of the key points. The connected key points are the 2D pose. In the detection-based approach, a CNN predicts a set of heatmaps for individual body parts. The fusion of the detected heatmaps gives the estimated 2D pose.

DeepPose by Toshev and Szegedy [11] was the first successful 2D human pose estimation approach that formulates a pose estimation as a CNN-based regression problem (Figure 5A). It is based on AlexNet, with an output layer that consists of the two-dimensional coordinates of the joints. By learning the joint coordinates directly, DeepPose suffers from the inability to generalize. Therefore, instead of determining the exact joint positions, heatmaps are introduced that indicate the confidence for each joint (Figure 5B) [124]. In addition, Tompson et al. [124] are jointly training a convolutional network for heatmap prediction and a graphical model, which allows to preserve geometric relationships between the joints of the body. However, a superior performance was achieved by a “stacked hourglass” networks algorithm [125], which did not use a graphical model. The idea of stacked hourglass networks is that spatial relationships on smaller and bigger scales are equally important for determining the human pose by combining information from different scales (Figure 5C). The basic building block of the proposed network is an hourglass module. There are three main components: the encoding, the decoding and the bypass. The encoding procedure uses convolutional and max-pooling layers to encode the information in the picture in decreasing resolution. At each stage, another convolutional layer is applied and stored at the bypass without pooling, so it remains in the same dimension as the layer pre-pooling. After reaching the minimum resolution (4 × 4 pixels), the decoding procedure begins. The network is symmetrical, so, for each decoding layer, there is an encoding equivalent. The process of combining information at two resolutions was described by Tompson et al. [124]. The architecture is called stacked hourglass because multiples of these modules are stacked behind each other. These stacked hourglasses produce a set of heatmaps, representing the probability of the presence of a joint at each position in the image. The output produced by the network is the estimated pose as the maximum activations across each heatmap.

#### 4.3.2. Pose Estimation in Three Dimensions

**Figure 6:**
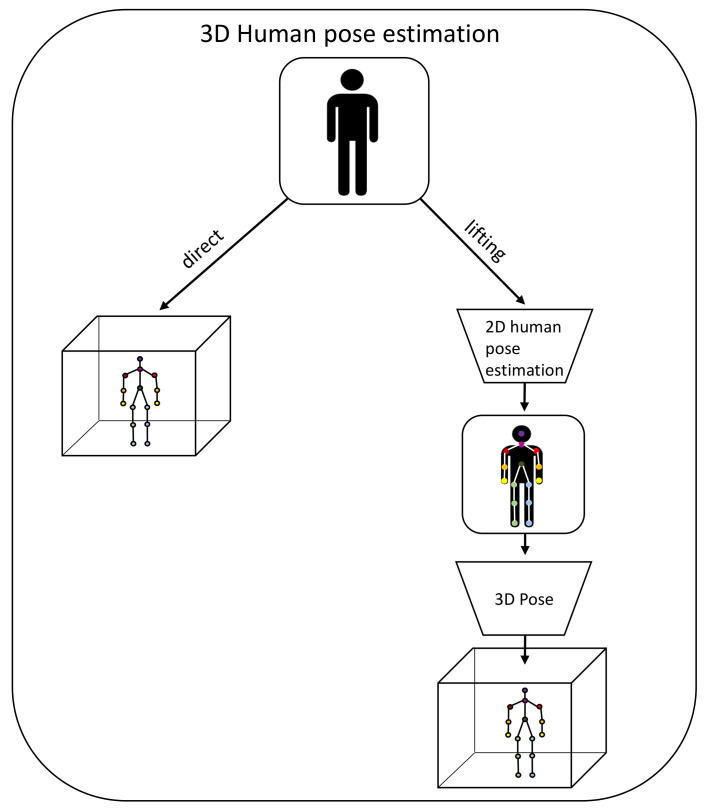
Schematic overview of 3D human pose estimation approaches with deep learning on an image. Two different approaches can be distinguished: direct and lifting.

Two-dimensional pose estimation refers to the estimation of the joint position in the two-dimensional picture, but the underlying human motions take place in a three-dimensional environment. In order to analyze the 3D motion, methods to infer 3D coordinates for the joints in a 2D image are necessary. These 3D coordinates are usually used with respect to one root joint that serves as the origin (e.g., the hip). Other types of encodings were discussed by Li and Chan [126]. Algorithms that approach the 3D pose estimation problem can be sorted into two categories [123]: 2D-to-3D lifting and direct estimation (Figure 6). In the direct estimation approach, the three-dimensional joint locations are directly inferred from the image without an intermediate step in the two-dimensional space [127]. The 2D-to-3D lifting approach identifies the joints in a two-dimensional image space first and then estimates the three-dimensional coordinates from the 2D joints [128,129]. In this way, the second approach benefits from excellent existing 2D pose estimation algorithms.

Human pose estimation techniques can be categorized according to input and output datatypes (Table 2). As we can see, 3D pose estimation techniques have been developed for many different input data types. RGB images or videos are the most common sources of materials. Many datasets are openly available [80,130,131,132]. Depth images provide the natural advantage of directly containing 3D information, which makes them perfectly suited for a task in three dimensions. Multiview images are most often generated in a controlled lab environment. Information about the camera setup can be used to enhance the estimation. Although they might not be suited to make pose estimations in real world applications, they might be a great first step to more complex experimental setups. Despite great advancements of 3D pose estimations, recent works have argued that some limitations should still be resolved before the extensive application of pose tracking for movement sciences is possible. Examples of limitations are the lacking estimation of important quantities such as accurate velocity and acceleration estimates; lacking the quantification of external forces; lacking estimates of the mass, size and inertia; biased demographics of databases and lacking the detection of contact or partial occlusions [12].

### 4.4. Action Recognition

Action recognition is usually defined as a classification task of matching an observed movement with a label. Action labeling can be performed for video (or image) data directly or for a sequence of joint coordinates estimated with the methods in Section 4.3. Some sensor technologies also provide sequences of 3D coordinates directly (see Inertial Measurement Unit in Section 5.3).

Some confusion and discussions have evolved around the terminology of action recognition. In this paper, we stick with the distinction as made by Moeslund et al. [118], which defined action primitives, actions and activities. Action primitives are defined as the most elementary motions, which are combined to form an action. When playing tennis, an action primitive could be “run”. Actions are more complex movements combining multiple primitives, like returning a ball in tennis. The activity is the broadest category—in this example, playing tennis. When training a novice, action recognition can be used to identify their actions and the order of execution of these actions. In order to provide feedback, the actions themselves, as well as their sequences, can be compared to the expert data with the methods described in Section 4.2. The next sections describe state-of-the-art methods to perform action recognition either from series of 3D coordinates for each joint or based directly on video input.

#### 4.4.1. Graph-Based Neural Networks

Graph convolutional networks (GCNs) are a recent neural network architecture that can use graphs as input [149], and GCNs have been used for action recognition successfully. Methods utilizing GCNs obviously need the movement represented as a graph. Popular encodings are spatiotemporal graphs [150,151,152]. Usually, the graph structure is a description of the skeleton structure, where each node represents a joint, and the edges indicate that two joints are connected by a limb. Movement data can be stored as either 3D coordinates for each joint and timeframe or as rotations around each joint from one timestep to the next. This data structure has the advantage of being very small, such that even large databases with movement data can be stored easily. It is also universally usable, as one can use data from inertial measurement units as well as image data to produce the graphs. A special case is the dynamic skeleton [153]. In addition to the edges representing the skeleton structure, it contains another set of edges: the temporal edges that connect the same joints in consecutive frames. Yan et al. [153] developed a Spatial Temporal Graph Convolutional Network (ST-GCN) that used this data structure for action recognition. Other methods based on GCNs do not consider the intraframe edges and instead infer the dependencies from the data. One such method is Attention-Enhanced Graph Convolutional LSTM (AGC-LSTM), which uses long short-term memory units to model spatiotemporal relationships between the frames [154]. Cho et al. [155] showed the importance of the appropriate design of the self-attention network for the performance of action recognition. The self-attention mechanism (SAN) also seems to be an important key for a better representation of spatial features of the human skeleton [156]. While many methods use the whole skeleton of the human body as a single graph, there are also approaches that consider part-based graph convolutions. In these methods, the human skeleton is divided into subparts (e.g., legs, torso, etc.). The network can then analyze the subparts first and aggregate the results to infer relations between them [157,158]. Datasets that are widely used for action recognition with GCNs are Kinetics-Skeleton [159], HDM05 [160] and NTU-RGB+D [77]. NTU-RGB+D contains 56,000 action clips from 60 different action classes. Each action clip is captured by three cameras with three different views. It also contains two different benchmarks: cross-view (CV) and cross-subject (CS). In the CV benchmark, the camera viewpoints are different. The training dataset consists of 37,920 action clips captured from cameras 2 and 3, and the test dataset consists of 18,960 action clips captured from the first camera. In the CS benchmark, the actors in the training and the test datasets are different. Table 3 shows the progress of state-of-the-art methods in action recognition with the aforementioned methods on the NTU-RGB+D dataset.

#### 4.4.2. Learning Directly from Video

In contrast to the graph-based methods, there are also methods that infer the action label directly from video data without intermediate processing like pose estimation. This might be a better solution if the data is collected as RGB and/or depth videos.

Image analysis can be done effectively with convolutional layers. These layers apply a filter to an image, which can learn to do edge detection or other useful operations. To process videos (stacks of images), a third (time) dimension can be added to the filter. Networks utilizing these layers for action recognition were introduced in 2010 [161]. Many improvements have been made to classification accuracy, as well as speed [162,163,164]. While these architectures produce state-of-the-art results, other developments in deep learning might be more fitting when focusing on motor learning. That is because the results from these networks are hard to understand or explain in hindsight. The user gets a good prediction for the action label, but it is difficult to tell why the network decided this way.

The idea to use two parallel networks for action recognition was introduced by Simonyan and Zisserman [165]. The goal is to separate the spatial and the temporal dimensions first and combine them only at the very end, when making a prediction. This was inspired by nature, where the human visual cortex is hypothesized to send visual information to two separate streams: the ventral stream (object recognition) and the dorsal stream (motion recognition). This was the inspiration of the two-stream network [165]. More information, e.g., sound can be added via new streams [166,167,168]. The architecture was further investigated by trying different ways of fusing the layers and deeper networks [169,170,171,172]. To facilitate the high computational costs of 3D convolutional layers, Lin et al. [173] introduced the Temporal Shift Module (TSM) that can be incorporated into 2D CNNs to model the exchanges among neighboring frames while maintaining the lower computational costs of 2D CNNs. To take different frame rates in videos into account, a frame–number-unified strategy can be applied on the temporal stream [174]. Recurrent networks and their extensions can be used to recognize actions on longer video sequences (>0.5 s) [175]. The approach to have the different aspects on different streams is interesting in motor learning examples, because it allows to trace errors back. In an assembly task, for example the information from the spatial stream would tell whether the worker stands in the right pose for the task, while the temporal stream might detect a wrong sequence of actions or a timing mistake. Two widely used datasets for action recognition on video data are the UCF-101 [176] and HMDB-51 [177] datasets. UCF-101 contains 13,000 annotated videos with 101 action classes. HMDB-51 consists of 6800 videos with 51 different actions. Table 4 shows the progress of the state-of-the-art methods in action recognition with the aforementioned methods on the UCF-101 and HMDB-51 datasets. The comparison also shows that the performance of an action recognition method strongly depends on the data it is trained with and does not necessarily generalize well.

Besides action recognition using video or using joints coordinates data, action recognition can be achieved by relying on motion data in the frequency domain. Several studies have converted human motion to the frequency domain using different methods [178,179] and used this additional information in the frequency domain for action recognition [180,181] or even for autoencoder-based motion generation [182]. Action recognition using information in the frequency domain also allows for faster performances, as compressed videos would be sufficient instead of regular RGB videos [181].

### 4.5. Motion Prediction

Using neural networks, not only pose estimation and action recognition drastically advanced, but pose prediction also became possible. This will undoubtfully become important for the interaction of machines with humans. For example, it will help to improve the safety of autonomous cars when they can predict well how the surrounding humans are likely to move [183]. Additionally, for safe human–robot collaborations, human motion prediction is necessary [184]. In addition, human motion prediction might also help to improve motor skill learning. For example, imagine executing a complex manual task involving several tools and material pieces during which you are assisted with virtual reality instructions. If the system can predict your motion well, it can detect faster if you are moving your hands to the wrong object or location and provide correctional instructions or give a quick warning to the user.

Different approaches have shown to be promising in the field of motion prediction. Bütepage et al. [185] developed a general representation of human motion that can be used as a generative model and as a feature extractor. They trained three different temporal encoders on a generic motion capture database to learn a low-dimensional representation of human motion dynamics. The resulting encoder–decoder models were successfully used for classification and prediction. This model is useful if one wants to use not only the prediction itself but also the extracted features for further analysis. In variations of encoder–decoder networks like the skip-attention encoder–decoder framework, the encoder is used to recognize the observed motion and the decoder to predict the following motion [186]. A second approach is using generative adversarial networks (GAN). A generative adversarial network consists of two models that compete with one another: the generative model generates new examples of a given data type, while the discriminative model tries to determine whether the new examples are real or fake [187]. Barsoum et al. [188] developed a GAN with a custom loss function designed for human motion prediction. A generative model predicted sequences of possible future human poses; simultaneously, a discriminative motion quality assessment model was trained to learn the probability that a motion sequence is a real human motion [188]. The generative model can produce not just one but many plausible upcoming movements with a corresponding probability. This probability estimation is useful to quantitatively assess the quality of the motion prediction and can thus prevent the occurrence of false instructions or warnings in a system for motor skill training. A third approach depends on residual neural networks. For instance, Martinez et al. [189] developed a sequence-to-sequence architecture with residual connections to predict human motion. They noticed a few disadvantages in previous residual neural networks to predict human motion, such as first frame discontinuity, hyperparameter tuning of a noise schedule and the depth and complexity of the networks. The following solutions were proposed: sequence-to-sequence architecture with sampling-based loss, a residual architecture and multi-action models. Three main experiments were performed to quantify the impact of these solutions, and they showed that their architectures outperformed previous residual neural networks. Analogous to the part-based approach from graph-based neural networks, Liu et al. [190] suggested using local GANs for different body parts and combining the results by using a global GAN. The methods described above performed different solutions to solve the human motion prediction problem.

### 4.6. Robot Motor Learning for Understanding Human Motor Learning

In this section, we will briefly describe how research in robot motor learning can expedite the research in human motor learning. Machine learning algorithms that are mainly used for robot motor learning are based on imitation learning, (deep) reinforcement learning, transfer learning or a combination of these [191,192,193]. Imitation learning or learning from demonstration is the task of teaching human behavior to a (humanoid) robotic agent [194]. In order to teach a robot a human motion, a demonstration of the motion needs to be recorded as a video or joint sequence (inertial measurement units). The sequence of features relevant for the task then needs to be extracted from the demonstration. This sequence of features needs to be learned by the robot. In reinforcement learning, the agent learns a new motor skill by trial-and-error, maximizing the reward function [191]. Finally, transfer learning is used to adapt a pretrained model in a training domain to a different test domains [195].

The algorithms described above can be helpful to solve an issue in the modeling of motor learning. Caramiaux et al. [191] pointed out that machine learning for movement modeling did not address enough the motor learning aspects, i.e., the adaptability of the movement to fine-grained changes (motor adaptation) and to radical changes (motor skill acquisition). In their study, they identified three prominently used adaptation categories in machine learning-based robotic motor learning: (1) parameter adaptation in probabilistic models (e.g., Hidden Markov Model or Dynamic Bayesian Networks)—useful for motor adaptation, (2) transfer and imitation learning—faster learning of new skills and (3) adaptation through reinforcement learning—improving stability in unstructured environments. They concluded that a combination of these would be a promising approach for motor learning models to be integrated into motor learning support systems. In this way, robot motor learning can be seen as a testbed for developing new human motion models. In summary, it is not only our understanding of human motor skill learning that remains limited but also the development of more intelligent robot motor control algorithms that adapt robot motions to changes and acquire new skills [191]. It is to be expected that progress in robot motor learning can boost progress in understanding human motor (skill) learning and vice versa [196].

## 5. Sensor Technologies for Human Motion Analysis

Several sensors are useful to study human motion, and an overview of some suitable sensors is given here. We divide technologies according to sensor type or device: RGB, depth and inertial sensors and virtual and augmented reality devices. Besides the discussed technologies, many others exist (e.g., marker-based motion capture, indoor GPS and stretchable and wearable electronics) but are not included in this brief overview, since it would distract this manuscript from its main focus.

### 5.1. RGB Camera

With the advancement of deep learning, the markerless detection of body parts via RGB cameras has become accurate and robust [9,10,11,197]. If using a stationary setup, it is often desirable that no sensors are worn on the body, so that motion is allowed to occur naturally [198]. Simultaneously registering human motion and detecting objects allows to study hand–object interactions [199] or helps to recognize actions or object affordances (i.e., functionality) [200] or to detect interaction hotspots between hand motions and objects [201]. With RGB cameras, it is possible to simultaneously detect the motion of multiple persons [10,135] or to investigate human–human interactions in a RGB video [202]. Vision is easy to scale up for the pose estimation of larger groups of people simultaneously or many people moving past a specific point [10,137]. Three-dimensional pose estimation is becoming increasingly accurate using only RGB data [134]. An RGB camera can be integrated in head-mounted virtual or augmented reality devices, allowing to detect one’s own hand motion and simultaneously registering the surroundings. Another advantage is the cheap hardware. Eye tracking is also possible with an RGB camera [203]. A deep learning approach to track eye motion in RGB data could make eye tracking available to anyone with a tablet or smartphone [204]. However, RGB cameras also bring their limitations, such as the occurrence of partial or complete occlusions of body parts [135] or the occurrence of conditions where the detection algorithms fail to detect motion (e.g., because of intense lighting or covered skin). Furthermore, unbalanced datasets to train algorithms result in a detection bias for different populations [205]. Despite the great potential for motor learning research, only a few studies used RGB data so far in human movement sciences. Cornman et al. [206] recently used pose estimation to assess finger tapping, and pose estimation was also used on online videos to study walker synchronization [207] and for gait analysis [208,209,210,211]. For an in-depth discussion of the advantages and limitations of pose estimation algorithms for movement sciences, we refer to Seethapathi et al. [12].

### 5.2. Depth Camera 

Different technological solutions (e.g., structured light, time-of-flight and coded aperture) can generate a depth image as the output [212,213,214]. Depth images have been used for body [215], hand [146,147,216,217] and object [218] pose estimations; simultaneous hand and object detection [219] and for action recognition [220]. The advantages of depth images for hand motion analysis are their robustness to change in shape, skin and size. In addition, depth sensors can easily be integrated into head-mounted devices, allowing to register the depth of the surroundings. A disadvantage is the susceptibility to ambient infrared sunlight. Fewer data are available for depth compared to RGB images, but data availability might change in the future, since depth sensors have become more frequently integrated into smartphones. Occlusions are still possible and can result in the failure of algorithms or inaccuracies. Most of the algorithms will need 3D models for proper 3D pose estimations, but these models are not always available. For hand pose estimations, this is not a big problem, since hand models can be easily generalized to different hands [8].

### 5.3. Inertial Measurement Unit

An inertial measurement unit (IMU) consists of an accelerometer and gyroscope, which measure acceleration and orientation, respectively, at one position on an object or a body. Additionally, an IMU can contain a magnetometer that allows to measure the heading with respect to the Earth’s magnetic field. To obtain accurate position data, sensory integration between IMU and GPS data is necessary, since IMU position data alone suffers from large integration drifts [221]. Inertial sensors allow to accurately detect motion, independent of the presence of visual occlusions. Sensors are often integrated in smartphones, which allows them to detect leg or arm motions with devices that many people already possess. However, sensors should always be worn on the body, which could restrict or interfere with movement, such as when a motion detection suit is too large or too small or when data gloves cover a person’s fingertips, reducing touch information.

### 5.4. Sensor Fusion

Each of the different sensors to gather motion data comes with its own advantages and limitations, which makes it hard to find the right option for an application. Therefore, researchers began to use multiple sensors and combine the data to overcome the limitations of the single techniques. Chen et al. [121] provided a review of papers combining RGB video, depth sensors and inertial sensors. Another example of sensor fusion for pose estimation is the work of Von Marcard et al. [222], who combined multi-view RGB video with inertial measurement units to improve the performance of a video-only estimation. They argued that, by combining very few inertial sensors (five in this case) with video data, they can overcome the limitations of both techniques. IMUs need a lot of set-up time and suffer from positional drift. On the other hand, the IMUs provide information where the multi-view video often fails, like the estimation of orientations for rotation-symmetrical limbs [222]. A similar approach was tested by Huang et al. [223]. When developing movement training applications, researchers may want to look for more than one technique to achieve the best results possible.

### 5.5. Virtual and Augmented Reality Devices

Virtual and augmented reality devices make use of sensor fusion, as they often combine many sensors into one mobile setup, which allows to combine the advantages of the different sensors. With virtual reality devices, the users are completely immerged in a virtual environment, while, with augmented reality devices, interaction with the real world remains possible, as well as with virtual objects. Both virtual reality and augmented reality can be valuable research tools. The advantages of virtual reality are the well-controlled experimental setup and increased ecological validity. It is possible for subjects to move in all directions; to track a subject’s hand, head and eye motions and to provide stimuli in relation to a subject’s position with high precision [224]. A limitation of virtual reality is the susceptibility of subjects to motion sickness [225]. The advantages of augmented reality are the ability to give virtual feedback in the real environment or during interactions with physical objects and tools. This way, trainees can practice for a new task and train the corresponding sensorimotor skills without an onsite trainer or coach [24]. It can allow the user to focus on the task at hand without having to shift focus to an external display, and it allows to stream video data and obtain instructions from a remote party [22]. A potential risk is that users become reliant on the virtual feedback; therefore, it might be useful to reduce the amount of information at specific points in the training process to prevent this dependency. As with any stereo imaging device, prolonged use could result in visual discomfort [226]. Future work in training with AR devices should focus on capturing skill performances and adjust instructions accordingly. In addition, if enough data from experts can be obtained, they could be used to develop and continuously refine an AR training system [24].

## 6. How to Transfer Motor Learning Principles to Complex Real World Environments?

In this section, we present different ways in which the technological advancements could support the transfer of insights about motor learning to real world environments to develop assistance systems for motor skill training. An example of such an assistance system could be a setup in which a surgeon gets feedback from augmented reality glasses while doing a surgery. Table 5 provides an overview of some recent existing studies that applied machine learning algorithms and/or recent sensor technologies to motor (skill) learning or motor behavior assessments.

**Figure 7:**
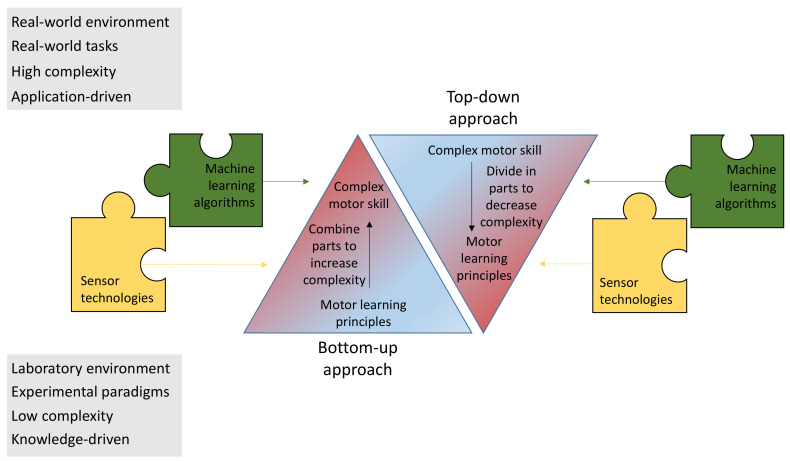
Two approaches are proposed: the left triangle shows the bottom-up approach, starting from motor learning principles measured with laboratory paradigms; the complexity is gradually increased by combining the known motor learning principles. The right triangle shows the top-down approach with a gradually decreasing complexity by dividing the complex motor skill into parts of lower complexity.

We see two major approaches to trigger progression in developing assistance systems for motor skill training. The first important approach, which the techniques discussed in Section 2, Section 3, Section 4 and Section 5 can achieve, is to scale up the motor learning principles from laboratory experiments to 3D real world problems. Currently, complex motor behavior has been largely left unexplored, since most studies are performed in well-controlled lab environments [27], but the surgeon in our example works in a three-dimensional body, using both hands and multiple tools. Gradually increasing the complexity of the studied motor behavior becomes possible with improved observation and analysis techniques. We call this first approach the bottom-up approach (Figure 7, left) since this approach starts from fundamental motor learning principles traditionally measured in a lab environment with well-controlled experimental paradigms. In this approach, the complexity is increased gradually to obtain a better understanding of complex motor skill learning. In contrast, in the second approach, one starts from a complex motor skill and gradually divides the motor skill into components of decreasing complexity to implement knowledge from motor learning principles to improve training systems, here called the top-down approach (Figure 7, right). In the bottom-up approach, the starting points are experimental motor tasks of lower complexity, while, in the top-down approach, the starting point is a complex real world motor task. The definition for ‘a complex motor skill’ is still under debate [241,242], but we here describe ‘complex motor skills’ as motor tasks with an infinite number of solutions to execute them. Due to the higher complexity of these tasks, it generally takes longer to train a complex real world motor skill [5] (e.g., hours, weeks or months) compared to the motor task of lower complexity in a laboratory environment. The bottom-up approach is knowledge-driven, as it starts from a research question. The top-down approach is application-driven, as it starts from a real world problem that requires a solution. Both approaches are useful to transfer the knowledge from motor learning to complex everyday motor skills and, hence, close the currently existing gap between motor learning research and real world motor skills.

### 6.1. Bottom-Up Approach: Improve Understanding of Motor Learning Principles That Are Relevant for Motor Skill Learning

Most motor learning paradigms are confined to well-controlled laboratory tasks with a strictly controlled number of trials, specified timing, controlled movement and accompanying reward for successful motion. The advantage of a controlled environment is the ability to study fundamental motor principles, apply specific manipulations and establish causality. A clear disadvantage is the ignorance of the complexity of the real world environment, with multiple degrees of freedom in the body movements and with multiple spatial and temporal solutions to a single task [243]. A bigger variety of paradigms is necessary to cover the whole range of natural real world motor learning [30]. This need for additional behavioral studies has also been emphasized for the overall research discipline of neuroscience and not only for the smaller subdiscipline of motor learning [244]. The algorithms and technologies discussed in Section 3 and Section 4 allow us to invent more diverse paradigms that closer resemble the real world environment.

The challenge of real world motor learning was recently addressed in a range of studies by Haar et al. and Campagnoli et al. [230,238,245]. The studies by Haar et al. developed an embodied virtual reality environment that allowed natural unrestricted body motion while, at the same time, controlling the experimental variables. The motor task was a pool game with the performance quantified with the trial error, the angular difference between the ball movement direction and the desired direction. The decay of error over the trials indicated that learning was achieved during the task. The recent study by Campagnoli et al. investigated the effect of 3D perception on explicit and implicit motor adaptation using a virtual reality environment [239]. Their findings suggest that explicit and implicit learning may rely on different sources of perceptual information, but they also stressed that more work is required to detect how depth cues influence the different learning principles.

**Figure 8:**
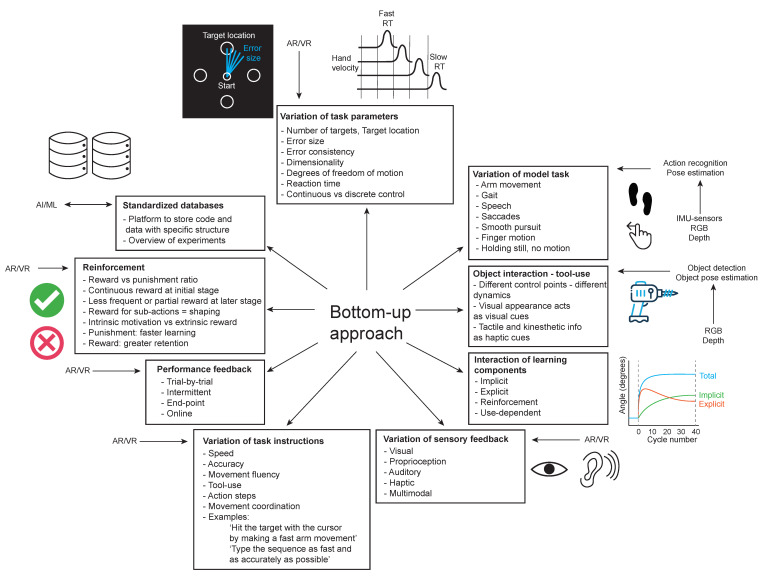
Bottom-up approach: improve the understanding of motor learning principles that are relevant for motor skill learning (VR: virtual reality, AR: augmented reality, ML: machine learning and AI: artificial intelligence).

Many insights into motor learning have been gained by dividing motor tasks into basic components of reduced complexity (e.g., implicit, explicit learning, use-dependent learning and reinforcement learning) [7,29], which has been a very successful approach to better understand motor learning and should definitely be continued for fundamental understanding. Nevertheless, we here suggest that, for the purpose of research transfer, the direction of increased complexity should be explored as well. A good start would be to gradually increase the motor task complexity to better match the diversity of real world motor behavior, such as adding the third dimension to the motor task [238,239], allowing unconstrained movement [246], investigating tool use [247,248] or increasing the task training time [249]. Increasing the complexity can be done at many levels; here, we propose some options (Figure 8). At several of these levels, recent developments in sensor technology (VR: virtual reality, AR: augmented reality), in machine learning (ML) or in artificial intelligence (AI) can create opportunities.

1. Variation of task parameters

The effects of many task parameters on motor adaptation of arm reaching are unclear so far. Overall, the early motor adaptation rate is higher with fewer targets (one vs. two vs. four targets). The relative contribution of explicit and implicit adaptations to the overall adaptation seems to be different depending on the number of targets, with a higher relative contribution of implicit adaptation with fewer targets [57]. Another study reported no effect of the number of targets on implicit adaptation (four vs. eight targets) [250]. Given these inconsistencies, it would be useful to further investigate the effect of the number of targets on explicit and implicit motor adaptations in future studies. Additionally, the target location could influence implicit motor adaptation, with higher levels of adaptation for diagonal compared to cardinal target directions [250]. The effect of target location on the explicit strategy has not been investigated so far, but as competition exists between explicit and implicit motor adaptations [251], the effect of the target location could be the opposite from implicit motor adaptation.

This approach to investigate the effect of the task parameters on motor adaptation is ongoing and should be continued for all possible task parameters: error size [250,252,253], error consistency [254,255], feedback timing [256,257], dimensionality [239], inter-trial time, movement speed, degrees of freedom of motion [258], reaction time [259] or continuous vs. discrete control [260].

2. Investigate a variety of model task paradigms

A lot of our understanding of motor learning comes from an arm reaching paradigm as the model task [7]. However, it is necessary to verify how these findings can be generalized to different movements. Besides arm reaching, many other movements can be explored: gait [42,43,44], speech [45,46,47], rapid eye movements or saccades [48,49,50], slower eye movement or smooth pursuit [51,52,53], finger motion [261] or the absence of movement in postural control of the arm and fingers [262]. Additionally, paradigms that consist of a combination of movements could be interesting, since motor skills are often a sequence or a simultaneous execution of different actions.

3. Investigate object interaction and tool use

Important for human motor behavior is the skillful interaction with tools or tool use. The motor system generates separate memories for different control points on an object if they are linked to different dynamics [247], even when the task implicitly defines these control points [248]. This study suggests that skillful interaction with an object or tool requires to consider the different dynamics of each part of the object. For accurate and calibrated motion, a human should thus not only have an internal model that represents its own body dynamics but also (internal) models that represent the dynamics of the objects with which the human interacts [39]. As most skilled motor tasks involve objects, motor skill learning will, to a large extent, also involve learning to control the dynamics of these objects. The visual appearance of these objects can act as cues providing information about the dynamics [263,264,265], and the same holds for tactile and kinesthetic information that can act as haptic cues for object dynamics [265]. As briefly touched upon during the overview of RGB and depth sensors in Section 4, besides human action recognition and human pose estimation, objects can also be detected in an image [13,266] and their poses estimated [8] or predicted [267]. Together with studying the human motor behavior, one can register the features of the objects with machine learning algorithms to come to a better understanding of the skillful interactions with these objects.

4. Investigate interaction of motor adaptation components

By taking the approach of dissociating motor adaptation in individual components, these components became better characterized. Nevertheless, it is useful to combine the components again to investigate the interactions between them. Depending on the experimental paradigm and the amount of each component, the balance between the different components fluctuates, as has been shown for explicit and implicit adaptation [268]. In a continuous reporting condition, verbal reporting and exclusion resulted in similar levels of assessed implicit and explicit adaptation, while, in the intermittent reporting group, verbal reporting resulted in more explicit and less implicit adaptation than in exclusion [268]. In addition, implicit and explicit adaptation are in competition in some contexts, with increases in the explicit system reducing the learning in the implicit system [251]. Besides implicit and explicit adaptation, several other components are known, like reinforcement learning or use-dependent learning [7,29]. As well as dissecting these components in additional subcomponents, for instance, implicit adaptation is driven not only by sensory prediction errors but also by target errors [269]. Upon discovering different components and subcomponents, the interactions between all of these can be determined to better understand complex environments where isolated components are scarce.

5. Investigate how different sensory feedback can modulate motor learning

In real world motor tasks, performance feedback can be given in diverse sensory modalities (for review, Sigrist et al. [270]): visual, auditory, haptic and multimodal. Visual information is the most straightforward feedback modality to induce motor learning. When learning a new motor skill, a more experienced person often shows the pupil how to perform the task [271,272]. This visual instruction serves as a reference of ideal task execution that the pupil can imitate. Besides visual instruction by an expert, pupils can improve their performance by observing each other [273], or visual feedback of one’s own motion can guide the learning process. Not surprisingly, several studies have investigated how visual feedback modulates motor adaptation. Tsay et al. [274] showed that visual uncertainty attenuated implicit motor adaptation, but it only did this for a smaller perturbation size. The visual uncertainty was simulated as a cloud of dots with a two-dimensional isotropic Gaussian distribution with a standard deviation of 10 degrees. However, in the small perturbation size, some of the dots induced errors of the opposite perturbation sign. In addition, a lower visual error consistency with opposite error signs also attenuated the level of the implicit component by downregulating the error sensitivity [255]. It remains thus unclear whether these error sign changes could have induced the differences between smaller and larger perturbations for attenuated implicit adaptation with higher visual uncertainty.

In most circumstances, visual feedback is much more reliable than proprioceptive feedback. In the dark or when vision is occluded, this changes. The effect of proprioception on adaptation is less well-investigated, presumably because of two reasons: (1) it is more difficult to control proprioceptive stimuli, and thus, causally investigating their effect on motor adaptation is harder as well, and (2) proprioceptive accuracy is lower than visual accuracy, and hence, the effects on motor adaptation are likely smaller. Nevertheless, several studies have investigated the effect of proprioceptive feedback on motor learning. A recent study [275] indicated that individual differences in proprioception could predict the extent of implicit motor adaptation, whereby increasing the variability and negative shift in proprioception, which was associated with higher levels of implicit motor adaptation. Future works should confirm the causality of this relation by manipulating the proprioceptive acuity experimentally, e.g., by perturbing the proprioception [276,277]. In addition, it could be of interest to simultaneously perturb the visual acuity and assess an individual’s proprioceptive acuity. By simultaneously assessing both sensory modalities, a better mapping between an individual’s implicit adaptation characteristics and sensory acuities can potentially be achieved. It remains to be investigated further how different sensory modalities interact during motor learning.

6. Investigate how different task instructions can modulate motor learning

The instructions in laboratory research are often well-standardized. For motor adaptation paradigms, participants are often instructed ‘to hit the target with the cursor by making a fast arm movement’ [54,55,278], while, in sequence learning, they are instructed ‘to type the sequence as fast and as accurately as possible’ [279,280,281]. In real world motor behaviors, instructions can be virtually anything, depending on the motor task. For more complex tasks, longer instructions are often required. For many motor tasks, instructions often specify how to manipulate certain tools or body parts.

7. Investigate how (sub)task performance feedback can modulate motor learning

In motor adaptation paradigms, the performance feedback is often given on a trial-by-trial basis. It could be end point feedback showing the accuracy of the reaching motion to the target or online feedback showing the reaching trajectory to the target [257,282]. Many other variations of feedback have been used in motor adaptation [261,283,284], and they seem to impact motor adaptation differently. It would also be interesting to investigate how performance feedback for different subtasks of a motor skill can impact the learning process.

8. Investigate how reinforcement can modulate motor learning

Reward and punishment differentially influence motor learning. Chen et al. [68] reviewed and discussed the effect of reward and punishment on motor skill learning. They concluded that novel laboratory-based motor skill paradigms should be developed to better assess the impact of reward and punishment on motor skill learning. In real world motor skill coaching, some practical guidelines for coaches exist: Reward and punishment should follow a ratio of about 80 to 20. In the initial stage of learning, continuous reward is beneficial, while, later in learning, less frequent, or partial, rewards would be better. Rewards should also be provided for improving sub-actions, called shaping. Finally, extrinsic rewards (like money or awards) can have different impacts on intrinsic motivations (i.e., the behavior itself is considered rewarding) [285,286,287].

Laboratory-based motor learning experiments have resulted in the following insights: punishment is leading to faster learning in motor adaptation, whereas reward is causing greater memory retention [62]. Reward is enhancing retention in a force tracking task [288]. Punishment resulted in faster reaction times in a serial reaction time task but impaired performance in a force tracking task [289]. Neither reward nor punishment improved memory retention in either the serial reaction time task or the force tracking task [289]. Finally, a stochastic reward benefited motor skill learning, boosting online gains and retention [290] potentially related to the positive effect of partial reward described in motor skill coaching.

In our opinion, a great potential exists for modern motion analysis algorithms and sensors to close the gap between the insights from (sports) coaching and laboratory-based motor learning experiments regarding the effect of reinforcement, as well as for improving the knowledge regarding the effect of instructions and performance feedback. For instance, virtual reality and augmented reality are great tools to provide and register standardized instructions, feedback and/or reinforcement; machine learning algorithms can potentially tailor feedback depending on the skill level and different motion sensor technologies can track a performance.

9. Create standardized collaborative database of motor learning experiments

Motor learning studies are increasingly sharing code and data online. A platform that refers to shared data from different studies is still missing. If data were stored in a predefined structure on such a platform, this could help generate a new hypothesis or testing models on existing data. This platform could also be used to store additional information regarding individual studies (e.g., hardware used, task instructions, reward specifications, target configuration, perturbation schedule and participant’s age). It could also help to get a better overview of the experiments conducted as the number of studies and complexity of the paradigms increases.

Many other aspects were left untouched in the scale-up levels presented above: repetition, attention, motivation, reaction times, eye gaze and coordination. The steps are by no means exhaustive and should simply be considered as a good starting point. For all these scale-up levels, recent developments in algorithms and hardware can play important roles to get closer to more complex and realistic motor tasks.

### 6.2. Top-Down Approach: Develop AI-Guided Assistance System for Motor Skill Training

**Figure 9:**
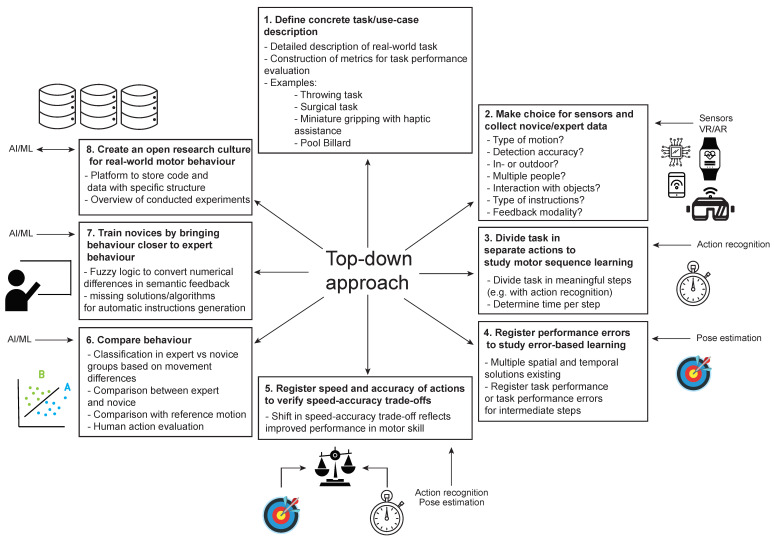
Top-down approach: 8 steps for making progress in developing an AI-guided assistance system for motor skills training (VR: virtual reality, AR: augmented reality, ML: machine learning and AI: artificial intelligence) (e.g., A: expert, B: novice).

In the top-down approach (Figure 9), we start from the motor skill and expert executions of that skill. We then apply machine learning algorithms and sensor technologies to train novices in that skill. This can be done by dividing the skill into sub-actions and give feedback on these smaller tasks to the novice. Feedback can be generated by machine learning algorithms that compare motions of experts and novices. An interesting question is whether we can target motor learning principles such as motor sequence learning or motor adaptation with the top-down approach. This could not only improve the training process of novices but could also develop a better understanding of motor learning principles applied in real world scenarios. In this context we already want to highlight that a prominent work in this direction is from the Nisky Lab, with a focus on surgical motor skills [231,291,292,293]. In the following, we will present an eight-step procedure that researchers can follow when applying the top-down approach (Figure 9). At several of these levels, recent developments in sensor technology (VR: virtual reality and AR: augmented reality), in machine learning (ML) or in artificial intelligence (AI) can create opportunities.

1. Define concrete task/use–case description

In the top-down approach, one starts with a particular skill of interest. This is usually a complex motor skill performed in real world scenarios. Recent examples are a throwing task [237] or a surgical task [231,292,293]. In the throwing study [237], 20 nonexpert right-handed participants performed overarm throws, starting from a fixed initial position. Participants were instructed to hit one of four circular targets positioned vertically at a 6-m distance; each target had a diameter of 40 cm. This study characterized the performance-related features of the high-dimensional motor task by a small set of indicators. These indicators could be used to distinguish the most skilled individuals and identify different strategies.

In Nisky et al. [292], the participants had to manipulate a surgical robot (da Vinci Si surgical system) using a custom-built grip fixture with their right hand. The task instruction was to move a virtual cursor dot from a starting position to a target as accurately and as quickly as possible. They developed metrics based on theories of motor control that allowed to assess the task performance for this very simple motion and detect improvements with practice blocks. This study showed how approaches from the field of motor control could be used to analyze motor behavior in a biomedically relevant application. In a perspective paper [293], Jarc and Nisky described how robot-assisted surgery could be used as an experimental platform to study complex motor skills in real world contexts. They argued that this platform would be beneficial since (1) both basic and complex tasks can be studied, (2) it can be extended to real world applications and (3) users with different levels of expertise exist for it. In a recent study [231], the performances of the participants was evaluated for a surgical needle driving task through artificial tissue under different haptic feedback conditions. They developed new metrics to evaluate the surgical needle driving task.

A third example is the steering and control of a miniaturized soft magnetic gripper with haptic assistance [294]. A micro-teleoperation system with haptic assistance for intuitive steering and control of a gripper in 2D space was developed. Two experiments with 26 human subjects showed that the system was viable, with significant improvements in the performance elicited by the haptic stimuli. The first task consisted of steering the soft gripper in a remote environment along a predetermined trajectory as fast and precisely as possible. The second task consisted of picking up a polystyrene microbead and dropping it off at a predetermined location while avoiding an obstacle along the path.

These examples (i.e., throwing, surgical robot operation and miniature gripping) show that an important first step in the top-down approach is a detailed description of the task or subtask of interest, and the construction of the metrics that can evaluate the task performance.

2. Make choice for sensors and collect novice/expert data

Selecting the right sensory system for a specific application can be a difficult task. First, it is important to keep the intended application in mind: What type of motion is being investigated (e.g., full-body, fine-scale hand or eye motion or large forces)? In which range should the motion be detected? Which detection accuracy is desired? Is the application intended for in- or outdoor use? Is the motion of a single person being investigated or are different people interacting simultaneously, or is there any interaction with objects, tools or with other digital devices? Should the users be provided with feedback or instructions, e.g., depending on their motion accuracy? Secondly, other parameters can constrain their choice: What is the available budget? Is the environment limiting the sensor choice (e.g., noisy, dusty or wet)? Do they prefer a fast setup time, fast calibration, easy usage, good user support, long battery life or low energy consumption? We did not intend to make a complete overview here to effectively select a specific, or a combination of, sensors or devices. Instead, we want to show that today’s possibilities are enormous, that they will continue to grow and that multiple solutions are possible. However, depending on the requirements for the application, the sensor selection can be constrained. In Section 4, we listed some sensors with their advantages and limitations, and this list can help to get started with the task of sensor selection.

3. Divide motor behavior in separate actions to study motor sequence learning

Motor sequence learning is often studied with a simple finger tapping paradigm where participants have to produce short sequences, often fewer than eight elements [31]. As indicated in the previous section, it would be beneficial to study sequence learning in more complex realistic behavior. This would require new techniques to assess the learning curve. Similarly, like in motor sequence learning studies in the laboratory, we could divide complex motor behavior into separate action steps.

Action recognition and pose estimation are useful techniques to divide this overall task into different meaningful action steps. For instance, action recognition has been used in manufacturing an assembly by Al-Amin et al. [26]. Their assembly example consisted of seven actions that could be recognized in multimodal sensor data. As the motor skill performance increases, the time to execute every single action is likely to decrease, since the time to execute a skilled action is often lower for experts than for novices [232] and so would be the overall execution time for a skilled task. We think this is like motor sequence learning, where a sequence of actions is executed faster with extended practice.

In addition, after separating the actions, other techniques such as pose estimation, full body motion tracking and eye tracking can be used to study in detail how individual actions become more efficient while learning a new motor skill. Moreover, individual actions likely consist of individual sub-actions that are optimized over time.

4. Register performance error to study error-based learning

Most motor learning research is performed in well-controlled lab environments, with very clear task instructions, separation of the task in different trials and two-dimensional movements. All these measures essentially reduce the variability to a minimum. This is in stark contrast with real world environments, where multiple spatial and temporal solutions exist to succeed in a single task [242,243]. Imagine two medical doctors performing the same type of surgery. They use a specific equipment set, use a specific motion pattern and work with a certain speed and applied force. Depending on the medical doctor, the entire procedure can be very different, although both surgeries might have a similar outcome. They might use different surgical techniques, which could be reflected in different eye motions and focus, or differences in hand and arm motions, either intended or corrective. Altogether, very different spatial and temporal solutions exist to solve the same task.

This redundancy, i.e., multiple solutions for the same task, makes error tracking not straightforward in real world motor tasks. A step-by-step detailed comparison of motion seems to be an unsolvable problem, given the many degrees of freedom in unconstrained variables. A good solution could be to also characterize the variations of these additional environment variables (e.g., type of equipment used and position and orientation of the tools), together with the movement. Given the large variability in solution spaces, a one-to-one detailed motion comparison between surgeons is not very insightful. Instead, group comparisons or individual-to-group comparisons make more sense after collecting larger datasets with multiple surgeons.

In motor learning research, a task performance error is defined as the mismatch between the task goal and the actual performance [253]. A task performance error can also be tracked in real world tasks, but often, a range of solutions exists. Instead of a single end task result, in a more complex task, one could also define several intermediate steps and register the task performance error for all these intermediate steps. These steps could be the separate actions that we discussed in the previous section. In motor adaption, the performance error is defined in degrees or applied forces, but when defining task performance errors for real world tasks, a variety of metrics will be necessary to track the performance during intermediate steps. When performing surgery, task performance errors might be a success or failure of the final surgical procedure. In music, it could be a difference in pitch or intensity. In dance, accuracy in timing, fluency of motion or posture. In sports, reaction times, speed of motion, posture or eye focus.

5. Assess speed–accuracy trade-offs of motor actions

In most behavioral tasks, a trade-off exists between speed and accuracy: the higher the speed, the lower the accuracy [295]. In most laboratory experiments, speed–accuracy trade-offs can be controlled to some extent by controlling either the speed or accuracy [296]. In motor learning research, this is often achieved by constraining the speed of movement to a minimum speed. If the movement speed is below the minimum desired speed, then no points can be obtained for reaching a target accurately [54,278,297]. If sampling the performances in different movement speeds, a complete speed–accuracy trade-off function can be derived [298]. A shift in this speed–accuracy trade-off function after training reflects an improved performance in motor skills [75]. In addition, training-induced accuracy improvements at the same movement speed also reflect a beneficial shift in the speed–accuracy trade-off. Together, it shows the importance of registering both speed and accuracy to assess performance improvements for intermediate motor actions. Therefore, for a quantitative comparison between subjects, it is essential to either constrain the speed or accuracy.

6. Compare behavior between experts and novices during skilled tasks

Nisky et al. [292] compared experienced robotic surgeons and novice users performing movements during the teleoperation of a da Vinci Si Surgical system and freehand (no manipulator). They showed that novices partially learned to adapt their movements to the dynamics of the robot manipulator, while experienced surgeons might already have an internal representation of the robot’s manipulator dynamics. This paper was the start of a range of studies by Nisky investigating the surgical motor skills for novices and expert surgeons. Recently, an optimal control theoretical framework was used to analyze differences in the task performances between novices and experts in a fine bimanual task (watchmaking) [232]. Coordination patterns between the hands were evaluated using three kinematic metrics (manipulability, task compatibility and comfort), and inverse optimization was used to infer optimal criteria. The differences in coordination patterns between novices and experts are interpreted as an alternation in the central nervous system’s optimal criteria accompanied by the learning process. The comparison of experts’ and novices’ behaviors during the execution of skilled motor tasks will help us to better understand how humans perform and learn skilled activities. In addition, comparison of the motions between groups [299] or with a desired reference motion [16] will allow to evaluate the motion and to give automatic and/or personalized instructions on how to improve the performance.

Finally, in recent years, human action evaluations have emerged as another field in human activity analysis with machine learning/artificial intelligence algorithms [300]. The aim of this field is to design computational models and evaluation approaches for automatically assessing the quality of human actions. It is thus not merely the recognition of actions or an estimation of human poses but, particularly, a quality assessment of how those actions were performed. In these novel algorithms, networks are often trained with experts’ ratings to estimate the skill level, which requires domain experts to provide the ground truth annotations.

7. Train novices by bringing behavior closer to expert behavior

Data collection from experts and novices for a motor skill could result in a classification of both groups based on movement differences [301,302]. This brings up the question of whether it would be possible to train the novices by giving them instructions that can reduce these differences. Patrona et al. [16] provided an interesting approach on how to train novices to correct their motor behaviors. They analyzed motion capture data by first detecting specific actions and comparing these actions to a reference motion. This required spatiotemporal alignment between the detected and reference motions. For the spatial alignment, they first normalized the bone length to compensate for body structure differences; next, they spatially aligned the data by correcting for the rotational offset of torsos. After this, the motion sequence was temporally aligned using multivariate dynamic time warping. Finally, the 3D positions of eight limb joints (i.e., elbows, wrists, knees and ankles) were compared with the reference, providing joint error statistics. These error statistics were fed into a fuzzy logic engine to produce semantic feedback, providing information on how to improve the action performance for the most erroneous joints.

In addition, performance evaluations in sports using wearable inertial sensors were enabled by a wide variety of criteria, such as technique analysis, spatiotemporal analysis, body and object orientation and action classification. Monitoring these aspects can potentially enhance training designs by the optimization of training stimuli and identification of training needs and opportunities [20]. We argue here that motor skills in general can benefit from evaluations based on such performance criteria, quantified with wearable inertial sensors but also other types of sensors (Section 4). Experts in a specific motor skill will allow to set desired or reference performance criteria and these criteria could help to establish targeted training of a motor skill.

Given the improvements in vison-based automatic skill assessment [300] and in performance evaluations with wearable sensors [20], automatic skill training or feedback systems are becoming increasingly feasible to develop. Nevertheless, new solutions are required to generate efficient and understandable instructions automatically; perhaps, methods applying the principles of explainable artificial intelligence can provide an answer [303].

8. Create an open research culture for real world motor behavior

When tracking motor behavior in more natural and complex conditions, datasets might become more diverse. Given this diversity, and for many other reasons, it is recommended for researchers to adhere to an open research culture by sharing their anonymized data and codes on repositories. Additionally, the preregistration of hypotheses and main analyses will be useful to better distinguish hypothesis-testing and hypothesis-generating research [304]. In addition, an online platform that provides a structured overview of the available studies with shared data and codes could be helpful to boost research progression. It could help to get a quick overview of the conducted experiments, to evaluate new analysis algorithms, to pretest hypotheses, to help design future experiments and to increase the data size for a specific motor task or a specific participant group. This overview platform could also list details of the experimental methods and materials (e.g., hardware, participants, instructions, task description, reward, assessed and controlled movement parameters).

## 7. Conclusions

The gap between motor learning in the laboratory and motor skills in the real world remains big. In this paper, after introducing a selection of concepts in motor learning, human motion analysis algorithms and sensor technologies, we suggested a two-fold approach to bridge this gap. The first is a bottom-up approach, starting from the motor learning principles and moving towards motor skill learning. The second is a top-down approach, starting from the motor skill of interest and dividing it into less complex components. For the bottom-up approach, we described several steps to gradually expand the existing lab experiments further to approach closer to more complex motor learning, where the error landscape is far more diverse and where the number of relevant variables is higher. A combination of human motion analysis algorithms and recent technological hardware developments allows to scale up the current motor learning principles to real world applications. In the top-down approach, we described different steps that could be useful to improve the training of motor skills. In these steps, sensors and machine learning algorithms can play a central role as well. Besides the top-down/bottom-up approaches, other developments could help advance the intended research transfer to real world scenarios. For instance, some questions that come to our minds: Are the current machine learning algorithms sufficient to support motor skill training? Can we design new machine learning algorithms that are better-suited to translate the detected differences between novices and experts into understandable and efficient training instructions? Will explainable artificial intelligence [305] provide these new algorithms, as its purpose is to make AI behavior more understandable to humans by providing explanations? Despite these unsolved questions, we foresee a bright future for the expansion of knowledge about motor skill learning and for the development of applications to train motor skills with improved efficiency of the training process.

## Figures and Tables

**Table 1:** Short summary of the similarity measures used to analyze human motion data.

Year	Authors	Data Type	Task
2018	Coskun, Tan et al. [108]	3D joint positions	Find better similarity measures between movements suitable for deep learning applications
2018	Guo and Wang [109]	3D joint positions	Motion Segmentation
2018	Li, Liu and Tan [110]	3D joint positions	Motion Segmentation
2018	Park and Kim [111]	3D hand positions	Find start and end of different tasks within a recording
2018	Pham, Hochin [112]	3D curves	Similarity between movements considering speed
2018	Sedmidubsky, Elias et al. [113]	3D joint positions	Machine learning based searching of large mocap databases
2018	Xia, Sun et al. [114]	3D joint positions	Motion Segmentation
2018	Zia, Zhang et al. [104]	Mixed	Comparison of clustering algorithms for surgical data
2019	Sedmidubsky, Elias et al. [115]	3D joint positions	Find subsequence in longer recording
2020	Pham, Hochin et al. [116]	3D curves	Compare sub-movements within a sequence
2020	Piorek, Jablonski [106]	Quaternions	Similarity measure based on rotations
2021	Aouaidjia, Bin et al. [117]	3D joint positions	Quantify similarity between movement sequences
2021	Park, Cho et al. [107]	RGB video	Compare two short video clips

**Table 2:** A collection of recent advances in human pose estimations. We show different application scenarios, depending on the data available and output desired.

Input Datatype	Output Datatype	
	3D	2D
RGB images	[127,133,134]	[135,136,137,138]
RGB videos	[122,123,139,140]	[9,141,142]
Multiview	[143,144,145]	
Depth images	[146,147,148]	

**Table 3:** Recent advances in action recognition with graph-based neural networks (GCN: Graph convolutional network, ST-GCN: Spatial temporal graph convolutional network, AGC-LSTM: Attention enhanced graph convolutional long-short term memory and SAN: self-attention mechanism). Accuracy is given for the action classes from the NTU-RGB+D database for two different benchmarks: cross-view (CS) and cross-subject (CS).

Year	Author	Method	Accuracy (NTU-RGB+D)
2018	Yan, Xiong and Lin [153]	ST-GCN	81.5% (CS)/88.3% (CV)
2018	Thakkar and Narayanan [157]	part-based GCN	87.5% (CS)/93.2% (CV)
2019	Li et al. [150]	ST-GCN (routing)	86.9% (CS)/92.3% (CV)
2019	Si et al. [154]	AGC-LSTM	89.2% (CS)/95.0% (CV)
2020	Cho et al. [155]	SAN	87.2% (CS)/92.7% (CV)
2020	Zhang et al. [156]	SAN -ST-GCN	96.9% (CS)/99.1% (CV)

**Table 4:** Recent advances in action recognition in video data. Accuracy is reported for action recognition on video data from the UCF-101 and HMDB-51 datasets.

Year	Author	Accuracy (UCF-101)	Accuracy (HMDB-51)
2014	Simonyan and Zisserman [165]	88.0%	59.4%
2015	Wu et al. [166]	92.6%	-
2016	Wang et al. [171]	94.2%	69.4%
2017	Li et al. [163]	92.5%	69.7%
2018	Lin, et al. [173]	95.5%	73.5%
2019	Crasto et al. [172]	95.8%	75.0%
2020	Kalfaoglu, et al. [162]	98.7%	85.1%

**Table 5:** Examples of recent studies in motor (skill) assessments with methods that apply machine learning and/or recent sensor technologies for human motion analysis.

Year	Authors	Motor Task/Motor Learning Principle	Integration of Motor (Skill) Assessment with…
2018	Butt et al. [227]	Catheter insertion	Virtual reality, haptics gloves
2018	Meyer et al. [228]	Juggling	Augmented reality; Ball and hand tracking
2019	Sharma et al. [229]	Prosthesis training	Augmented reality
2019	Chambers et al. [207]	Human gait	Pose estimation in YouTube videos
2020	Stenum et al. [210]	Human gait	Pose estimation (with OpenPose)
2020	Haar, van Assel, Faisal [230]	Pool billard	IMU motion tracking suit
2020	Bahar et al. [231]	Robot-assisted needle driving	Haptic feedback in virtual environment
2020	Yao and Billard [232]	Watchmaking, Bimanual fine manipulation	Hand pose estimation; modeling (inverse optimization)
2020	Harris et al. [233]	Golf putting	Virtual reality, motion tracker on real golf club
2020	Vanneste et al. [234]	Product assembly	Augmented reality
2020	Ropelato et al. [235]	Ophthalmic microsurgery	Augmented reality
2021	Lilija et al. [236]	Precise hand motion	Virtual reality
2021	Tommasino et al. [237]	Ball Throwing	Dimensionality reduction techniques
2021	Haar, Sundar, Faisal [238]	Pool billard	Embodied virtual reality
2021	Campagnoli et al. [239]	Visuomotor rotation	Virtual reality
2021	Zhang and Sternad [240]	Ball throwing	Virtual reality
